# Correlates of Suicide Ideation and Attempt among Youth Living in the Slums of Kampala

**DOI:** 10.3390/ijerph9020596

**Published:** 2012-02-16

**Authors:** Monica H. Swahn, Jane B. Palmier, Rogers Kasirye, Huang Yao

**Affiliations:** 1 Institute of Public Health, Georgia State University, P.O. Box 3995, Atlanta, GA 30302, USA; Email: janepalmier@gamail.com (J.B.P.); sawsinnj@gmail.com (H.Y.); 2 Uganda Youth Development Link, P.O. Box 12659, Kampala, Uganda; Email: kasiryer@yahoo.com

**Keywords:** suicidal ideation, suicide attempt, street youth, homeless youth, vulnerable youth, adolescents, sadness, loneliness, parental neglect, trading sex, Africa, Uganda, Kampala

## Abstract

While suicidal behavior is recognized as a growing public health problem world-wide, little is known about the prevalence and risk factors for suicidal behaviors among street and slum youth in Africa, and in Uganda, specifically. The number of youth who live on the streets and in the slums of Kampala appears to be growing rapidly, but their mental health needs have not been documented, which has hampered resource allocation and service implementation. This study of youth, ages 14–24, was conducted in May and June of 2011, to assess the prevalence and correlates of suicidal behavior. Participants (*N* = 457) were recruited for a 30-minute interviewer-administered survey through eight drop-in centers operated by the Uganda Youth Development Link for youth in need of services. Bivariate and multivariate logistic regression analyses were computed to determine associations between psychosocial correlates and suicide ideation and suicide attempt. Reporting both parents deceased Adj.OR = 2.36; 95% CI: 1.23–4.52), parental neglect due to alcohol use (Adj.OR = 2.09; 95% CI: 1.16–3.77), trading sex for food, shelter or money (Adj.OR = 1.95; 95% CI: 1.09–3.51), sadnesss (Adj.OR = 2.42; 95% CI: 1.20–4.89), loneliness (Adj.OR = 2.67; 95% CI: 1.12–6.40) and expectations of dying prior to age 30 (Adj.OR = 2.54; 95% CI: 1.53–4.23) were significantly associated with suicide ideation in multivariate analyses. Parental neglect due to alcohol use (Adj.OR = 2.04; 95% CI: 1.11–3.76), sadness (Adj.OR = 2.42; 95% CI: 1.30–7.87), and expectations of dying prior to age 30 (Adj.OR = 2.18; 95% CI: 1.25–3.79) were significantly associated with suicide attempt in multivariate analyses. Given the dire circumstances of this vulnerable population, increased services and primary prevention efforts to address the risk factors for suicidal behavior are urgently needed.

## 1. Introduction

Every year, almost one million people die from suicide; a global mortality rate of 16 per 100,000, or one death every 40 seconds [1] and as such it is a critically important public health problem [2]. Suicide is the fourth leading cause of death globally among youth 15 to 19 years of age [3] and the tenth leading cause for adolescents 10–14 years of age [4]. However, these figures do not include suicide attempts, which are up to 20 times more frequent than completed suicide [1]. Research on suicidal behavior specifically in Africa has been scarce [5,6], in part because of other pressing health concerns, but also because of political and economic instability [7]. 

Suicidal behavior in Africa was thought to be rare in the past, but recent studies suggest that it represents a substantial public health burden [5,6]. Studies conducted in Nigeria [8], South Africa [9], Zambia [10], and Uganda [5,11] indicate that suicidal behavior is relatively common, but also varies across countries [12]. For example, among school students, self-reported suicidal ideation ranges between 19.6% in Uganda, 23.1% in Botswana, 27.9% in Kenya, and 31.9% in Zambia [12]. Alcohol use and bullying victimization [12], as well as experiencing hunger [13], appear to be important risk factors for suicidal ideation. However, the high levels of suicidal ideation and behavior among youth in sub-Saharan Africa have likely been exacerbated by the severe psychosocial stress and other adverse health outcomes associated with the high prevalence of HIV/AIDS in Africa [2] as well as scarce food supply [14] and other distressing circumstances. In addition, the stigma, discrimination, isolation, lack of support from family and friends, loss of parents or family members from HIV/AIDS further increases the risk of suicidal behavior [7].

Youth who live on the streets and in the slums of sub-Saharan Africa face much of the burden related to poverty, lack of family support as well as infectious and chronic diseases [15,16]. However, there is a dearth of research on the specific health needs of these vulnerable youth and their strategies for coping and trying to survive under such dire circumstances. It has been noted that street children face complex issues, but also that more information is needed for resource allocation and the design and implementation of prevention and intervention efforts [15,16]. The health concerns of street children in Kampala is a particularly pressing issue because they are expected to substantially increase in numbers as Uganda is projected to have the world’s highest population growth over the next couple of decades [17].

Depending on the age groups and definitions used, there are between 100 million and 300 million street youth worldwide, most of them in low- and middle-income countries [18,19,20]. Most of the world’s street children are boys, although estimates of girls living on the streets range from one-fourth to one-third worldwide [21]. Throughout the world, poverty is the underlying cause of the high number of street children [21,22]. Substance abuse, mental and physical health problems among street youth appear to be a substantial problem and the provision of scarce medical and mental healthcare presents challenges to organizations working with this vulnerable population [19].

There is a dearth of information regarding the specific mental health needs and concerns among street youth in sub-Saharan Africa, but it is expected that youth who live on the streets and in the slums have high levels of suicidal ideation and behavior. Research from other countries may not be generalizable to sub-Saharan Africa, but may provide relevant context. A few studies of suicidal behavior among street youth in the U.S. and Canada have found that the prevalence of suicide attempt ranges between 20% and 40% [23,24] and that suicide was a leading cause of death in this population [25]. Abuse, whether physical, sexual or other victimization [23,24] is a key risk factor for suicide risk as is family substance abuse [23,26], youth substance use [23], social stigma [27], depression [23,24], low self-esteem and feeling helpless [28]. The underlying conditions for these problems have been attributed to abusive caretakers and to street culture which encourages high-risk behaviors such as alcohol and drug use, prostitution, stealing and drug dealing [24].

Given the dearth of research of the scope of mental health needs and concerns among youth who live on the streets and in the slums in sub-Saharan Africa, this study examines the prevalence and risk factors for suicidal ideation and attempt specifically among such youth in Kampala, Uganda. The purpose of this project is to obtain quantitative data to assess demographic characteristics, family context, psychosocial factors such as substance use, sexually transmitted diseases including HIV/AIDS, sadness and loneliness and their associations with suicide ideation and attempt in a convenience sample of service-seeking youth. Findings from this study can be used to quantify the scope of unmet needs in this vulnerable population and to seek resources and strategies to implement prevention and intervention strategies.

## 2. Methods

The overarching goal of the cross-sectional survey called the “Kampala Youth Survey”, conducted in May and June 2011, was to quantify and describe high-risk behaviors and exposures in a convenience sample of urban youth living on the streets or in the slums, 14–24 years of age, who were participating in a Uganda Youth Development Link (UYDEL) [29]; drop-in center for disadvantaged street youth. UYDEL serves on average about 650 youth per month through these drop-in centers. Face-to-face surveys, lasting about 30 minutes, were administered by social workers/peer educators employed by UYDEL. The study was implemented across eight drop-in centers across Kampala. Participating youth received snacks and transportation for completing the survey. No identifying information was collected and the surveys were completely anonymous. Surveys were administered in English or Luganda, to the extent possible, in private settings and rooms, to ensure privacy of survey questions and responses. 

Each social worker/peer educator received training on the study methodology, each of the survey questions and its translation into Luganda (local language) if needed, and recruited potential participants among attendants at their specific drop-in Center. Recruitment took place using word-of-mouth, and each attendant was eligible for participation if they were between 14 and 24 years of age. No exclusion criteria were applied beyond the age range. Participants were informed about the study and read (or were read) the consent forms to indicate their willingness to take the survey. The consent process required that emancipated street youth 14 to 17 years of age provide their own consent for participating in the survey (Because youth 14 to 17 years of age who “cater for their own livelihood” are considered emancipated in Uganda, parental permission/consent had been waived.) The same consenting process was followed for youth 18 to 24 years of age. 

Over the ten-day survey period, 507 youth were approached for participating in the survey. Among these youth, 46 declined and 461 agreed to participate, yielding a participation rate of 90.9%. Four of the surveys were missing substantial numbers of responses and were therefore excluded, yielding 457 completed surveys for the final analytic sample of youth between the ages of 14 and 24 (31.1% boys and 68.5% girls). The mode for age was 17 years (*n* = 81) and 67% of participants were between ages 16 and 20.

### 2.1. Measures

The survey questionnaire was modeled from existing surveys such as the U.S. based Youth Risk Behavior Survey [30] conducted by the Centers for Disease Control and Prevention and the international Global School-based Student Health Survey [31] supported by the World Health Organization and which provides data on health behaviors and relevant risk and protective factors among students across all regions served by the United Nations. The study development was also informed by training materials related to working with street children prepared by the World Health Organization [32]. Survey questions addressed demographic characteristics, family context, alcohol and drug use, injuries, violence and suicidal behaviors, sexual behaviors and sexually transmitted diseases, including HIV/AIDS. 

The study protocol was approved by the Georgia State University Institutional Review Board and also by the Uganda National Council for Science and Technology. Funding to conduct the study were obtained from the Georgia State University Office of International Initiatives and also from funds leveraged through collaboration with the Emory Center for Injury Control, funded by the Centers for Disease Control and Prevention. 

### 2.2. Analysis

The measures included in the analyses and their prevalence among study participants are described in Table 1. Bivariate and multivariate logistic regression analyses were computed to determine statistical association between suicide ideation and suicide attempt and demographic and psychosocial correlates using the SAS 9.2 and SUDAAN 10 statistical software packages. Moreover, all variables except for parental living were dichotomized to indicate the presence or absence of the particular risk factor. The reference category for each variable in the logistic regression analyses was the absence of the particular risk factor.

## 3. Results

The demographic characteristics and family context of the participants are presented in Table 1. With respect to family context, 76% of the participants had one or more deceased parent, 63% reported ever being hit or beaten by their parents and 21% reported that their parent’s alcohol use had prevented them for providing care for them. 

**Table 1:** Description of variables and their prevalence among youth living in the slums of Kampala (*N* = 457).

Descriptive label	Variable Wording	Percentage
School attendance	Percentage of youth who currently attend school	14.4%
Both parents dead	Percentage of youth with both parents dead	39.6%
One parent dead	Percentage of youth with mother or father dead	36.3%
Two parents living	Percentage of youth with both mother and father alive	23.0%
Parental physical abuse	Percentage of youth who reported that their parents ever hit/beat them (yes *versus* no)	62.6%
Parental neglect due to alcohol use	Percentage of youth who reported their parents’ alcohol use made them not able to care for them (yes *versus* no)	21.0%
Apprenticeship skills	Percentage of youth who state that they have any apprenticeship skills	56.0%
Any drug use	Percentage of youth who have ever used drugs such as marijuana (njaga or bangi) or opium (njaye or sniffed aviation fuel (one or more days)	13.8%
Any drunkenness	Percentage of youth who have ever been really drunk (one or more days)	32.6%
Any STD/HIV	Percentage of youth who have been told by a doctor or nurse that they have a sexually transmitted infection, such as syphilis, bolabola or gonorrhea, or that they have HIV/AIDS (yes *versus* no)	37.2%
Any traded sex	Percentage of youth who have ever gotten money, food, or other things for have sexual intercourse with someone (yes *versus* no)	31.7%
Any rape	Percentage of youth who have ever been forced to have sex with someone (yes *versus* no)	24.1%
Sadness	Percentage of youth who ever felt so sad or hopeless almost every day for two weeks in a row in the past year that they stopped doing their usual activities (yes *versus* no)	75.1%
Loneliness	Percentage of youth who have felt lonely in the past month (sometimes/often *versus* never)	81.8%
Expect to die early	Percentage of youth who think they will probably die before the age of thirty (sometimes/often *versus* never)	43.5%
Suicidal ideation	Percentage of youth who have thought of killing themselves in the past year	30.6%
Suicide planning	Percentage of youth who have thought about how they would kill themselves in the past year.	22.9%
Suicide attempt	Percentage of youth who tried to kill themselves in the past year.	19.8%
Suicide attempt requiring medical help	Percentage of youth who needed medical help after trying to kill themselves in the past year.	11.9%

In terms of schooling and skills, 14% were currently attending school and 56% reported that they had acquired some apprenticeship skills. Loneliness, sadness, and expectations of dying before age 30 were reported by 82%, 75% and 44%, respectively. 

**Figure 1:**
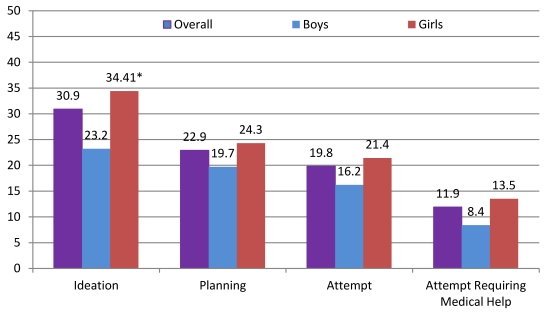
Prevalence of suicidal ideation and suicidal behaviors for boys and girls living in the slums of Kampala (*N* = 457). * Girls were more likely than boys to report suicide ideation; OR = 1.73; 95% CI: (1.10–2.73).

The overall prevalence of reporting suicide ideation (31%), suicide attempt planning (23%), suicide attempt (20%), and a suicide attempt requiring medical treatment (12%) and for boys and girls separately, are presented in Figure 1. The prevalence of suicide ideation was statistically higher for girls (34%) than boys (23.2%) (OR = 1.73; 95% CI: 1.10—2.73). However, no other significant differences were observed between boys and girls in terms of having planned or made a suicide attempt or having required medical treatment following a suicide attempt.

In the bivariate logistic regression analyses, having two deceased parents, reporting parental neglect due to alcohol use, any drug use, any drunkenness, any sexually transmitted diseases, any traded sex, sadness, loneliness, and an expectation of dying prior to the age of 30 were significantly associated with suicide ideation (Table 2). The psychosocial correlates that were significant in the bivariate analyses were entered into a multivariate regression analyses. The multivariate analyses show that having two deceased parents, parental neglect due to alcohol use, any traded sex, sadness, loneliness, and an expectation of dying prior to the age of 30 were significantly associated with suicide ideation (Table 2). 

In the bivariate logistic regression analyses for suicide attempt, reporting parental neglect due to alcohol use, any drug use, any drunkenness, any sexually transmitted diseases/HIV, any traded sex, sadness, loneliness, and an expectation of dying prior to the age of 30 were significantly associated with the outcome (Table 3). Again, the psychosocial correlates that were significant in the bivariate analyses were entered into a multivariate regression analyses. The multivariate analyses show that parental neglect due to alcohol use, sadness, and an expectation of dying prior to the age of 30 were significantly associated with suicide attempt (Table 3). 

**Table 2:** Bivariate and multivariate associations between suicidal ideation and demographic characteristics, family context and psychosocial correlates among youth living in the slums of Kampala (*N* = 457).

Variable	Suicide Ideation		
	%	OR (95% CI)	Adj. OR (95% CI) *
Sex (Girls/Boys)	23.62/7.28	**1.73 (1.10–2.73)**	**2.31 (1.26–4.23)**
School attendance	5.52	1.46 (0.85–2.52)	-
Both parents dead	9.33	**1.93 (1.15–3.23)**	**2.36 (1.23–4.52)**
One parent dead	10.89	1.21 (0.75–1.95)	1.37 (0.78–2.41)
Two parents living	10.22	1.00	1.00
Parental physical abuse	21.44	1.40 (0.91–2.16)	-
Parental neglect due to alcohol use	10.80	**2.49 (1.56–3.99)**	**2.09 (1.16–3.77)**
Apprenticeship skills	18.75	1.17 (0.77–1.77)	-
Any drug use	6.40	**2.17 (1.26–3.74)**	1.96 (0.90–4.28)
Any drunkenness	13.25	**1.95 (1.29–2.97)**	0.93 (0.49–1.75)
Any STD/HIV	13.85	**1.59 (1.06–2.39)**	0.65 (0.38–1.11)
Any traded sex	14.67	**2.66 (1.74–4.05)**	**1.95 (1.09–3.51)**
Any rape	8.07	1.12 (0.70–1.77)	-
Sadness	28.22	**3.30 (1.83–5.98)**	**2.42 (1.20–4.89)**
Lonely	29.53	**5.24 (2.33–11.78)**	**2.67 (1.12–6.40)**
Expect to die early	18.97	**2.72 (1.80–4.12)**	**2.54 (1.53–4.23)**

* Variables included in the multivariate logistic regression analyses are those significantly associated with suicide ideation in the bivariate analyses. All variables included in the adjusted analyses are listed in the table. Statistically significant associations are boldfaced. All variables except for parental living were dichotomized to indicate the presence or absence of the particular risk factor. The reference category for each variable was the absence of the particular risk factor.

## 4. Discussion

In this study of youth who live in the slums of Kampala, it is clear that the mental and physical health needs in this vulnerable population are of grave concern. The overall prevalence of suicide ideation (31%) was higher in comparison with nationally representative youth in Uganda (22%) [12]. The underlying factors for the increased burden of suicidal ideation and behavior may be best captured and understood in terms of the family context and the coping mechanism used to face the circumstances of living on the streets and in the slums of Kampala. 

First and foremost, more than three quarters of the youth in our survey reported one or both parents deceased, an alarming issue that is likely the key underlying cause of their current living conditions. In our study, reporting that two parents were deceased was significantly associated with suicidal ideation in multivariate analysis. This issue is not unique to the youth in Uganda, since it is estimated that approximately 13 million children in the world have lost either one or both parents to AIDS [2]. However, more than 90% of these children live in sub-Saharan Africa [2] which makes it a more pressing concern in this region. Moreover a substantial proportion of the youth in our study reported having been hit or beaten by their parents and also that their parents had been unable to care for them due to their alcohol use which was an important correlate of suicidal ideation and suicide attempt in multivariate analysis. Parental alcohol abuse is a key concern and a likely contributor to the current living situations for these youth and also because of the documented role of alcohol in child abuse and neglect [33,34]. This problem may be particularly salient in Uganda which has the highest estimated *per capita* consumption of alcohol use world-wide [35]. Trading sex for food, shelter and money and other risky sexual behaviors have been raised as a key concern among vulnerable street youth, not only in sub-Saharan Africa [36], but also elsewhere [37]. Reports of trading sex is typically associated with an urgency of finding food and shelter or money for basic needs and is more commonly reported among street children. In our study, about a third (32%) of the youth reported having traded sex. Moreover, traded sex was an important and statistically significant correlate of suicide ideation in the multivariate analysis. 

**Table 3:** Bivariate and multivariate associations between suicidal attempt and demographic characteristics, family context and psychosocial correlates among youth living in the slums of Kampala (*N* = 457).

Variable	Suicide Attempt		
	%	OR (95% CI)	Adj. OR (95% CI) *
Sex (Girls/Boys)	14.73/5.05	1.41 (0.83–2.38)	-
School attendance	3.74	1.53 (0.83–2.81)	-
Both parents dead	5.53	1.51(0.83–2.74)	-
One parent dead	7.08	1.16(0.67–2.00)	-
Two parents living	6.86	1.00	1.00
Parental physical abuse	13.93	1.48 (0.89–2.48)	-
Parental neglect due to alcohol	7.71	**2.76 (1.65–4.62)**	**2.04 (1.11–3.76)**
Apprenticeship skills	12.44	1.43 (0.86–2.37)	-
Any drug use	4.84	**2.60 (1.45–4.66)**	1.65 (0.78–3.47)
Any drunkenness	8.79	**1.97 (1.22–3.18)**	1.00 (0.51–1.95)
Any STD/HIV	9.19	**1.63 (1.02–2.61)**	0.89 (0.50–1.58)
Any traded sex	9.09	**2.12 (1.32–3.41)**	1.41 (0.77–2.56)
Any rape	5.13	1.11 (0.65–1.90)	-
Sadness	18.88	**5.19 (2.19–12.31)**	**3.20 (1.30–7.87)**
Lonely	18.93	**5.22 (1.85–14.76)**	2.56 (0.85–7.71)
Expect to die early	12.67	**2.72 (1.67–4.40)**	**2.18 (1.25–3.79)**

* Variables included in the multivariate logistic regression analyses are those significantly associated with suicide attempt in the bivariate analyses. All variables included in the adjusted analyses are listed in the table. Statistically significant associations are boldfaced. All variables except for parental living were dichotomized to indicate the presence or absence of the particular risk factor. The reference category for each variable was the absence of the particular risk factor.

The mental health needs of these youth were assessed through questions that addressed several related concepts, sadness (76%), loneliness (82%), expectations of dying (hopelessness) (44%), and suicide ideation, attempt and suicidal behaviors. Sadness, loneliness, and expecting to die early were statistically associated with suicide ideation in multivariate analysis. Sadness and expecting to die early were also found to be significantly associated with suicide attempt in multivariate analysis. Taken as a whole, these findings underscore great social isolation and despair and calls for more mental health services and interventions for these vulnerable youth. Previous research has shown that street children in Kenya, compared to controls, have high levels of adaptability and flexibility when faced with adversity [38]. However, there may be limits of flexibility because of the possible cumulative effect of victimizations and other adverse health factors that subsequently is expressed as sadness and isolation when there no longer is a social network available for support. 

The results of this study should be viewed in the context of several important limitations. First, the study participants were not randomly selected, but were youth who self-selected to attend the drop-in centers and to take part of the study. Therefore, the findings may not be representative of street and slum youth in Kampala and may not be generalizable to populations elsewhere. However, the high response rate in the targeted drop-in centers increases our confidence that the findings are representative of the youth who self-select to attend drop in centers or to seek services more generally. Second, the sample included both street youth who were homeless and youth who lived in the slums but may have had a stable living arrangement. Accordingly, our definition of youth who live in the slums was broad and included a range of circumstances and family contexts (e.g., some of these youth currently lived with two caretakers or in a treatment setting and were relatively well fed and cared for), although circumstances varied significantly. If the study had only included homeless street youth, or if we had been able to differentiate these groups better, the prevalence of the health risk behaviors as well as suicide ideation and attempt would likely have been significantly higher among homeless street youth specifically. However, all youth who participated in the survey were help-seeking youth in that they had participated in services or training classes provided by the UYDEL which may also indicate that these youth are not representative of street youth more broadly and may also at least partially explain why our study included mostly girls. One of the most frequently offered training class by the centers was hairdressing and braiding which is likely to be attended by girls. 

Third, because of limited literacy rates, participants were read the questionnaire. The face-to-face interaction between participant and interviewer may have resulted in underreporting of certain high-risk behaviors associated with significant stigma (such as transmission of sexually transmitted diseases or even suicidal ideation or behaviors). While this is an important potential concern because participants (who knew the interviewers) may have been unwilling to disclose sensitive information, it is less likely since the participants selected to attend the drop in centers and discussions of sensitive information was part of many of the services provided. Fourth, during the interviews, participants often provided the answers in Luganda (local language) which required the interviewers to translate the response to English and make the appropriate notations on the questionnaire. This issue was anticipated and discussed during the training of the interviewers and appeared to have been handled appropriately during the data collection by the interviewers who were all proficient and fluent in both spoken and written English. 

Fifth, most of the questions regarding mental health factors and high-risk behaviors were selected from previously established self-administered surveys, particularly the Youth Risk Behavior Survey [30] conducted in the U.S. and the Global School-based Student Health Survey [31] conducted primarily in Africa, Asia, and Latin America. In several cases, the wording of the questions used was simplified and the response options narrowed in order to facilitate the administration of the survey questionnaires by the interviewers. As such, the reliability and validity of some of the measures may have been altered. Sixth, because of the cross-sectional nature of the survey, the temporal ordering of the psychosocial factors and suicide ideation cannot be determined, nor can causation be inferred. Finally, there were fewer statistically significant correlates of suicide attempts than suicide ideation. This finding is most likely due to the increased severity of attempt *versus* ideation. However, this finding may also in part be attributable to the lower prevalence of suicide attempt among participants rendering those analyses less strong as indicated by the slightly wider confidence intervals.

**Figure 2:**
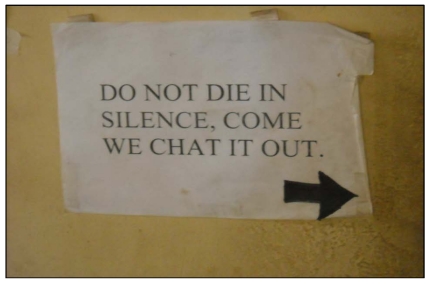
Note posted on wall in Drop-in Center, by UYDEL staff, to encourage help-seeking behaviors among potentially suicidal youth.

Despite these limitations, the findings clearly underscore grave and unmet needs among these vulnerable youth which has implications for research and practice. While these vulnerable youth may have unique circumstances and needs, overall the psychosocial correlates associated with suicide ideation examined in this study mirror those of previous studies of other African youth [8,10,12,39]. However, additional research is needed to replicate these findings since they are primarily based on a convenience sample of youth who have sought out services offered by a drop-in center. As such, their experiences may be different than that of other youth and should be documented in greater detail and also preferably in a longitudinal cohort to better determine the initiation of risk behaviors and the identification of modifiable factors that may be suitable for prevention. 

Future research is also needed to better determine strategies for providing additional services and treatment to these youths who may be difficult to reach. This is particularly troubling given the acute shortage of psychiatrists, psychologists, nurses, and social workers in Africa [40] and in Uganda, more specifically [41]. Moreover, future projection indicates that both self-inflicted deaths will increase substantially by the year 2030 [42] which further underscores the need to strengthen the infrastructure of mental health services and to add capacity, perhaps by incorporating mental health into primary care [42] and increasing the scope and training of lay workers [41]. The staff at UYDEL has sought to implement strategies to encourage help-seeking among potentially suicidal youth (Figure 2); however, more comprehensive efforts, resources and guidance will be needed to reach more youth.

In terms of the implication for practice, it is important to recognize that evidence-based strategies used elsewhere [43] may not be relevant for a low-income country such as Uganda or for vulnerable youth with particular needs and circumstances. That said, brief interventions and contact following suicide attempts which have been found to be effective for reducing subsequent suicide mortality [43] may possibly be adapted to local conditions and may even be applied in a primary prevention mode. Moreover, other strategies have been suggested for the development of child mental health services in low income countries [44] including school based mental health programs [45] that may also be promising for adaptation to be delivered to youth who seek services in drop-in centers. Recent findings have emphasized that child and mental health efforts begin with awareness and that schools in many countries are willing to support positive change to address mental health needs [46]. These strategies may also be adapted for other settings outside of schools that provide services to vulnerable youth.

Given the limited healthcare resources available for treatment and prevention of infectious and other diseases, it is clear that public health issues such as suicide and self-directed violence have been given less priority [47]. However, the findings from this study can be used to advocate the urgency for providing more resources and services to these vulnerable youth, most of which have lost one or more parents and have faced significant adverse childhood experiences. Because of the significant burden of these youth, their circumstances will further compound existing problems including the spread of sexually transmitted diseases. As such, resources need to provide basic shelter and services to them, but also seek ways to reduce the social isolation and hopelessness that they report and emphasize primary prevention of high risk behaviors. The priority of providing more services to vulnerable youth needs to be considered complimentary to, and not competing with, other prevention efforts underway to address HIV/AIDS and other critical health problems including malaria, hunger and poverty. Finally, as has been called for previously, it is important to create a national suicide prevention plan with local community support [48] so that future interventions, policies, and resource allocation can yield the impact needed and to improve the conditions and health outcomes of these vulnerable youth. 
